# Remibrutinib as a Therapeutic Option for Solar Urticaria

**DOI:** 10.1111/phpp.70127

**Published:** 2026-10-06

**Authors:** Joshua Mijares, Manoj Bhagwat, Johanna Gonzalez, Vignesh Ramachandran

**Affiliations:** ^1^ Department of Dermatology Indiana University School of Medicine Indianapolis Indiana USA; ^2^ Skin Institute of New York New York New York USA; ^3^ Prava Medical San Francisco California USA

**Keywords:** chronic inducible urticaria, remibrutinib, solar urticaria

To the Editor,

1

Solar urticaria (SU) is a rare, chronic form of inducible urticaria that manifests as rapid‐onset erythematous and pruritic wheals, often with a burning or stinging sensation, upon exposure to UV or visible light [1]. The pathogenesis of SU centers on IgE‐dependent mast cell activation triggered by solar electromagnetic radiation penetrating the skin and acting on a cutaneous chromophore [1]. The condition can substantially impair quality of life, limiting outdoor activity and causing systemic symptoms with extensive exposure [1]. Along with photoprotective measures, current guidelines recommend second‐generation H1‐antihistamines as first‐line therapy [2]. However, a substantial proportion of SU patients remain symptomatic despite these interventions, and options for antihistamine‐refractory disease are limited, including photohardening, omalizumab, cyclosporine A, IVIG, and plasmapheresis [2].

Remibrutinib, a highly selective oral Bruton tyrosine kinase (BTK) inhibitor, has recently demonstrated efficacy in the management of chronic spontaneous urticaria (CSU) by blocking mast cell and basophil degranulation downstream of FcεRI and other Fc receptors, thereby preventing the release of histamine and proinflammatory mediators regardless of whether activation is IgE‐ or IgG‐mediated [3, 4, 5]. Given this mechanism, remibrutinib may offer therapeutic benefit in other mast cell‐driven urticarias, including chronic inducible subtypes such as SU. Here, we report a case of long‐standing SU inadequately controlled by standard‐dose dual antihistamine therapy that achieved complete symptom resolution with remibrutinib in combination with cetirizine.

A 28‐year‐old female with Fitzpatrick skin Type I presented to the dermatology clinic with an 8‐year history of SU. She reported the onset of pruritic, well‐demarcated erythematous plaques with confluent erythema and subtle edema within 5 to 10 min of sun exposure, localized exclusively to sun‐exposed skin with sparing of photoprotected areas (Figure 1). Reactions were not accompanied by systemic symptoms such as headache, gastrointestinal upset, wheezing, or syncope. She had no personal history of atopic disease and no family history of SU, other physical urticarias, or autoimmune disease. The condition substantially limited her ability to engage in outdoor activities. Baseline complete blood count and comprehensive metabolic panel were within normal limits. Given the classic presentation, we did not pursue photoprovocation testing, action spectrum determination, minimal urticarial dose assessment, or further diagnostic workup for alternative photodermatoses (e.g., porphyria, cutaneous lupus erythematosus). The diagnosis was made clinically based on the characteristic history and morphology of her reaction.

**FIGURE 1:**
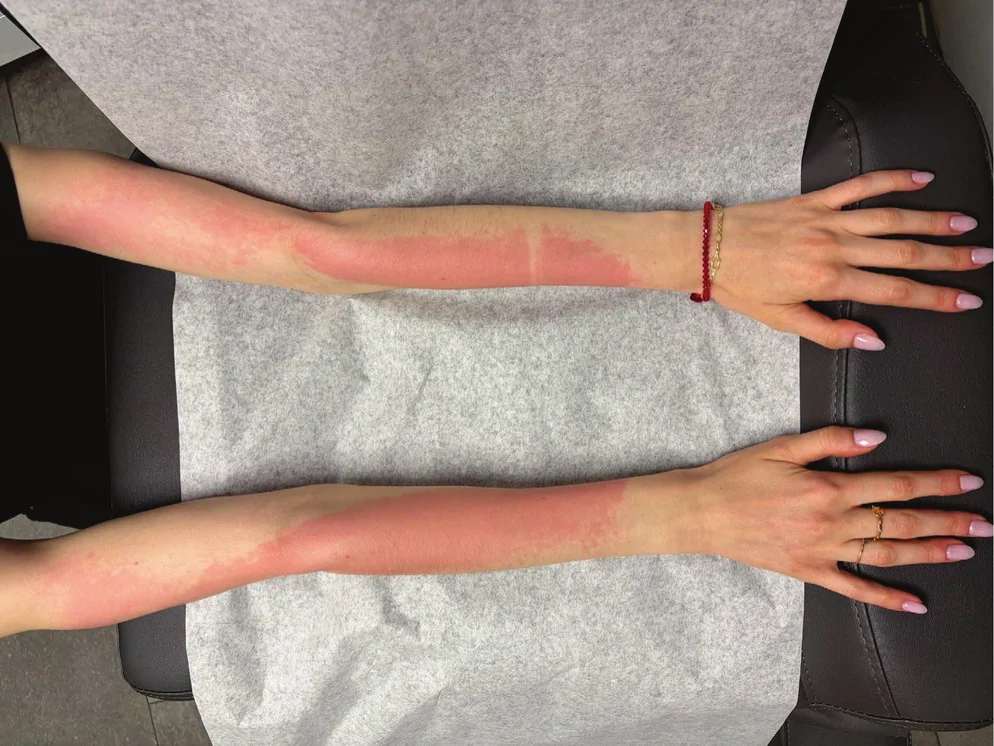
Photograph of the patient's arms and forearms immediately following 30 min of sun exposure prior to initiation of remibrutinib, demonstrating well‐demarcated, confluent, erythematous, urticarial plaques.

She was initially treated with cetirizine 10 mg daily and fexofenadine 180 mg daily along with gradual sunlight exposure for several years, which provided only minimal relief of pruritus and did not prevent urticarial reactions. Updosing of antihistamines was briefly attempted per current urticaria treatment guidelines but was discontinued due to sedation. Omalizumab was discussed but declined by the patient due to its black box warning of anaphylaxis and its injectable delivery method. A single dose of dupilumab 300 mg was subsequently administered concurrently with cetirizine and fexofenadine; however, the patient similarly preferred an oral medication and also desired a faster‐acting treatment option, so dupilumab was discontinued. Given dupilumab's characteristically delayed onset of action, this brief trial did not allow adequate time to assess efficacy [6].

Given inadequate symptom control despite antihistamine therapy, she was started on off‐label remibrutinib 25 mg twice daily in combination with cetirizine 10 mg nightly (fexofenadine was discontinued at this time). She noted subjective improvement with her first occurrence of sun exposure after treatment initiation and achieved complete resolution of symptoms within 1 week, with full tolerance of sun exposure thereafter (Figure 2). At 4 months of follow‐up, she has remained symptom‐free without breakthrough reactions, has required no dose adjustment or tapering, and has experienced no adverse effects such as bleeding, nausea, abdominal pain, headaches, or frequent upper respiratory infections.

**FIGURE 2:**
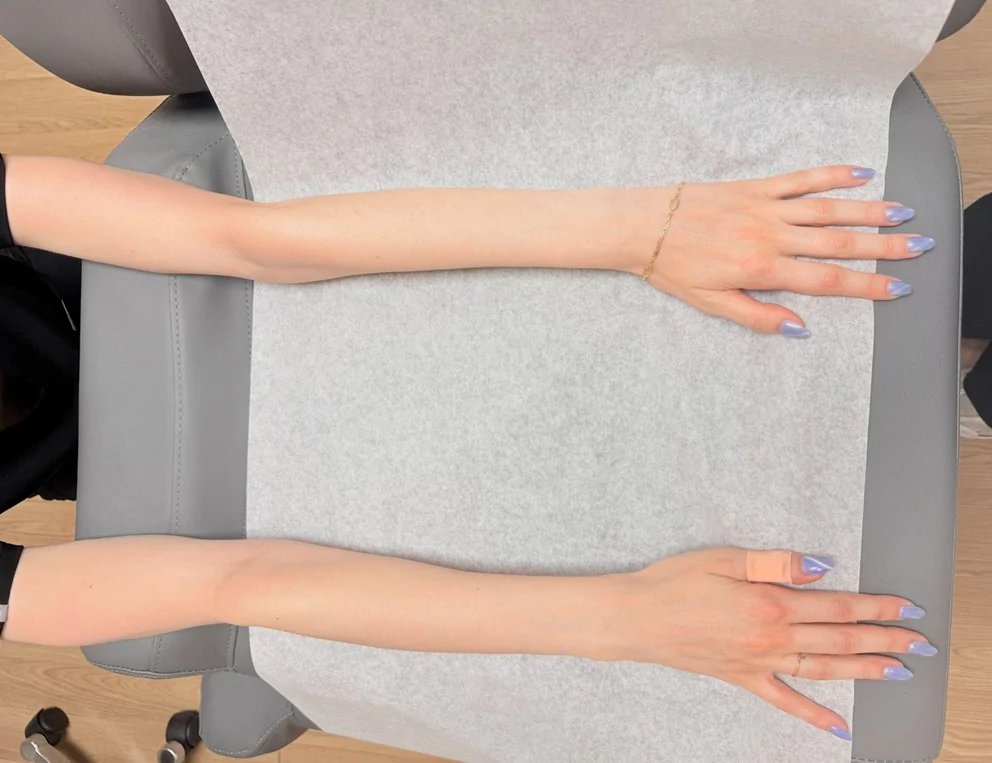
Photograph of the patient's forearm immediately following 30 min of sun exposure after 1 week of treatment with remibrutinib, demonstrating complete resolution of urticarial lesions with no evidence of erythema, edema, or wheals.

Our patient experienced improvement with her first occurrence of sun exposure after initiation and complete resolution within 1 week. Although direct comparison between this single uncontrolled case and published clinical trials is not possible, reported outcomes for established therapies provide context. For example, second‐generation H1 antihistamines, the first‐line treatment for SU, provide immediate symptom attenuation, but their efficacy is modest, with an overall response rate of 83% and a complete response rate of 7.7% [2]. Omalizumab, another off‐label treatment option, demonstrates the highest efficacy among available therapies, with a pooled response rate of 93.2% and a complete remission rate of 68.4%, but may require weeks to months to achieve maximal benefit [2]. The rapid and complete response observed in this patient supports the hypothesis that BTK inhibition can effectively suppress mast cell‐driven urticarial reactions independent of the specific upstream trigger (e.g., cutaneous photoallergens), extending prior observations in chronic spontaneous urticaria to a chronic inducible subtype. This mechanism would be further supported by additional data suggesting positive responses to remibrutinib in other chronic inducible urticaria (CINDU) subtypes, such as heat urticaria, cholinergic urticaria, or aquagenic urticaria.

This case adds to the emerging literature on BTK inhibition in CINDU beyond CSU, an approach with very limited prior reports. One case report recently described the first use of remibrutinib in SU, describing a 49‐year‐old man with refractory SU who similarly achieved complete symptomatic remission within 1 week of treatment initiation [7]. Our case independently corroborates that observation and extends it in three aspects: the response was sustained through 4 months of follow‐up; remibrutinib was given in combination with a standard‐dose second‐generation antihistamine rather than as monotherapy; and benefit was observed in a patient who had not received omalizumab and did not tolerate antihistamine updosing, which suggests that the efficacy of remibrutinib may extend to patients who have not exhausted established second‐line therapies.

Currently, all clinical trial data for remibrutinib in urticaria are limited to CSU [8]. An ongoing Phase 2 trial (NCT06865651) is now evaluating remibrutinib in multiple CINDUs [9]. Although the small sample size of 44 patients across multiple CINDU subtypes may limit the number of SU‐specific cases for analysis, these data may help establish whether the response observed in this case can be replicated in a controlled setting.

This report has limitations, including the lack of generalizability from a single case, a relatively short follow‐up of 4 months, the lack of omalizumab treatment, and the short timing of dupilumab usage. Additionally, the absence of baseline and post‐treatment photoprovocation testing and minimal urticarial dose measurement precludes objective quantification of the treatment response, which was instead assessed by patient‐reported symptoms and clinical photography alone. Nonetheless, the rapid and complete response observed here, in a patient with SU inadequately controlled by standard‐dose dual antihistamine therapy and who had an incomplete trial of dupilumab, offers a compelling rationale for evaluating BTK inhibition as a treatment strategy in SU.

## Funding

The authors have nothing to report.

## Ethics Statement

The authors have nothing to report.

## Consent

The patient provided written informed consent for the publication of this case report, including clinical information, laboratory data, and associated images.

## Conflicts of Interest

The authors declare no conflicts of interest.

## Data Availability

Data sharing not applicable to this article as no datasets were generated or analysed during the current study.
